# Syndecan-4 regulates extravillous trophoblast migration by coordinating protein kinase C activation

**DOI:** 10.1038/s41598-019-46599-6

**Published:** 2019-07-15

**Authors:** Mariyan J. Jeyarajah, Gargi Jaju Bhattad, Brianna F. Kops, Stephen J. Renaud

**Affiliations:** 1 Department of Anatomy and Cell Biology, Schulich School of Medicine and Dentistry, University of Western Ontario, London, Ontario Canada; 2 Children’s Health Research Institute, Lawson Health Research Institute, London, Ontario Canada

**Keywords:** Cell invasion, Mechanisms of disease, Developmental biology

## Abstract

Extravillous trophoblast (EVT) invasion is an essential component of human placentation. Poor EVT invasion is associated with obstetrical complications including preeclampsia. Integration of cues from the extracellular environment is required for directional EVT invasion, but how EVTs coordinate responses to these cues is not well understood. Syndecan-4 (SDC4) is a transmembrane heparan sulfate proteoglycan that binds to, and modulates the activity of, many extracellular proteins implicated in placental development. Therefore, we determined the functional importance of SDC4 for EVT invasion. We found that SDC4 is expressed by a first trimester EVT line (HTR8), and in EVTs in placenta throughout pregnancy, with higher expression during early pregnancy than at term. Higher expression was also observed in placentas from preeclampsia compared to normotensive pregnancies. SDC4-deficient HTR8 EVTs exhibited reduced migration and Matrigel-based invasion, both under basal conditions and following exposure to basic fibroblast growth factor and heparin-binding epidermal growth factor. SDC4-deficient HTR8 EVTs also showed reduced protein kinase C-alpha (PKCα) and AKT phosphorylation. SDC4 directly bound to activated PKCα in EVTs, and inhibition of PKCα decreased EVT invasion and migration. Our findings reveal an essential role of SDC4 as a regulator of EVT motility, in part through coordination of PKCα activation.

## Introduction

Extravillous trophoblast (EVT) invasion is an essential feature of human placentation. During the first half of pregnancy, invading EVTs emanate from the placenta, migrate into the uterus as far as the inner third of the myometrium, and infiltrate the uterine spiral arteries^1^. Initially, EVTs occlude lumens of spiral arteries, thereby hindering maternal blood flow to the placenta and causing the placenta to transiently develop in a low oxygen environment^2^. The EVT occlusions gradually dissipate, and cells progressively displace the endothelium, internal elastic lamina, and smooth muscle layers surrounding the spiral arteries. This process results in transformation of spiral arteries into dilated, flaccid conduits capable of delivering a large and consistent supply of maternal blood to the placenta for the remainder of pregnancy^1^. Several serious obstetrical complications are associated with insufficient EVT invasion and reduced spiral artery transformation, including early-onset preeclampsia^3,4^, intra-uterine growth restriction (IUGR)^4^, and spontaneous abortion^5^, highlighting the importance of EVT invasion for successful pregnancy.

A variety of extracellular signaling molecules (matrix components, growth factors, chemokines, proteases, and cytokines) have been identified that modulate EVT motility (reviewed in^6–8^). What is not well recognized is how EVTs integrate these distinct extracellular cues to activate intracellular signaling pathways, thereby facilitating tightly controlled migration during early placental development. Heparan sulfate proteoglycans (HSPGs) are cell surface and extracellular matrix macromolecules containing a core protein to which heparan sulfate glycosaminoglycan sidechains are attached. The heparan sulfate chains are capable of binding to, and modulating the activity of, more than 200 extracellular proteins^9^. By sequestering and concentrating these extracellular proteins, HSPGs can alter spatial distributions of growth factors, chemokines, proteases, and protease inhibitors, thereby facilitating the establishment, maintenance, and potentiation of signaling gradients. HSPGs also act as co-receptors for a variety of growth factors implicated in placental growth and development, including fibroblast growth factors (FGFs)^10^, epidermal growth factors (EGFs)^11^, vascular endothelial growth factors^12^, and family members of the Wnt^13^, bone morphogenetic protein^14^, and hedgehog pathways^15^. Additionally, HSPGs can themselves serve as signaling molecules when cleaved from the cell surface. Thus, HSPGs are multi-faceted coordinators of developmental signaling pathways^16,17^. Disruption of HSPGs leads to impairments in focal adhesion turnover, cell adhesion, and cell migration^18^, but their role in regulating EVT motility and placental development is not well known.

HSPGs are classified into several families depending on the structure of their core protein. Only one family of HSPGs, the syndecan (SDC) family, possesses a transmembrane domain enabling them to bridge extracellular interactions with intracellular signaling cascades. In mammals, SDCs are represented by four genes, *SDC1*, *SDC2*, *SDC3*, and *SDC4*, which encode proteins containing distinctive extracellular domains (ectodomains), a highly conserved single-pass transmembrane domain, and cytoplasmic domains that confer the ability to interact with intracellular scaffolding proteins and initiate a wide range of signaling processes^19^. In human placenta, only SDC1, SDC2, and SDC4 are detectable. SDC1 is predominantly expressed in syncytiotrophoblast (the outer trophoblast layer of chorionic villi), SDC2 is detected in all trophoblast and mesenchymal lineages, and SDC4 is highly expressed in invasive EVTs and villous cytotrophoblasts during early pregnancy^20,21^. Intriguingly, SDC4 is the only family member enriched in focal adhesions, where it forms a physical link between extracellular signaling proteins such as growth factors and matrix molecules, and intracellular scaffolding proteins and signaling molecules such as protein kinase C alpha (PKCα)^22^. SDC4-mediated PKCα activation initiates a series of downstream signals that promote focal adhesion assembly, Rho-GTPase and AKT activation, cytoskeletal reorganization, and directional cell migration. Consequently, cells deficient in SDC4 have abnormal morphologies and impaired migratory potential^23^.

Given the known importance of SDC4 for focal adhesion turnover, cytoskeletal organization, and cell migration, and high expression of SDC4 in EVTs, we postulated that SDC4-PKCα signaling promotes EVT invasion. In this study, we examined the importance of HSPGs for EVT invasion and used a knockdown approach to determine the role of SDC4 for EVT motility. Moreover, we show that SDC4 is dynamically regulated in placenta throughout pregnancy, and that aberrant placental expression of SDC4 is evident in early-onset preeclampsia.

## Results

### Heparin and Heparinase III attenuate EVT invasion and migration

**Figure 1:**
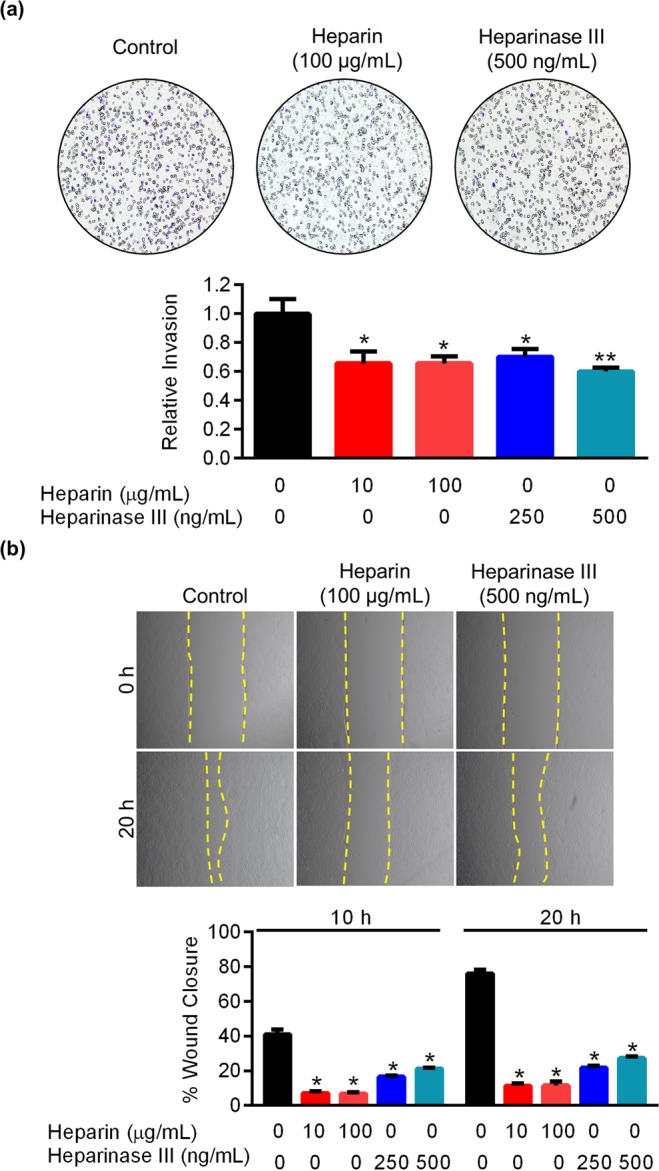
Heparin and Heparinase III attenuate HTR8 EVT invasion and migration. Matrigel-based invasion assays and scratch-wound assays were conducted using untreated HTR8 EVTs, and cells exposed to heparin (10, 100 μg/mL) or heparinase III (250, 500 ng/mL). (**a**) Number of HTR8 EVTs that invaded through Matrigel following exposure to heparin or heparinase III compared to untreated cells. Representative images of membranes with invaded cells are shown above the graph. (**b**) Percent gap closure by untreated HTR8 EVTs compared to cells exposed to heparin or heparinase III, 10 h and 20 h following introduction of a wound. Representative images of wounds at 0 h and 20 h are shown above the graph. Yellow dashed lines denote wound edges. Values significantly different from controls are indicated with an asterisk (*P < 0.05; **P < 0.01). Graphs represent means (SEM).

To determine whether HSPGs are necessary for EVT invasion and migration, Matrigel-based invasion assays and scratch-wound migration assays were performed using a well-established EVT line, HTR8/SVneo (henceforth referred to as HTR8 EVTs). Cells were treated with different concentrations of unfractionated heparin (which competitively inhibits heparan sulfate chains interacting with ligands^24^) or heparinase III (which cleaves heparan sulfate chains from HSPGs^25^) during assays. Compared to controls, approximately 35% less cells invaded through Matrigel following exposure to 10 µg/ml or 100 µg/ml unfractionated heparin (Fig. 1a, N = 4, *P* < 0.05). Similarly, the ability of cells to invade Matrigel was reduced following exposure to heparinase III (30% (*P* < 0.05) and 40% (*P* < 0.01) decreased invasion in cells exposed to 250 and 500 ng/ml heparinase III, respectively; Fig. 1a, N = 4). When analyzing scratch-wound migration assays, control cells migrated into the wound such that 41% of the total area was closed 10 h after the wound was introduced. In contrast, cells treated with 10 and 100 µg/ml unfractionated heparin closed the wound area 7% and 6%, respectively. Likewise, cells treated with 250 ng/ml and 500 ng/ml heparinase III also exhibited reduced migratory ability, closing the wound area by 16 and 21%, respectively (Fig. 1b, N = 4, *P* < 0.05). After 20 h, control cells filled 76% of the original wound area, whereas cells treated with 10 and 100 µg/ml unfractionated heparin closed the initial wound area by approximately 11%, and cells exposed to 250 and 500 ng/ml heparinase III closed the gap less than 25% (Fig. 1b, N = 4, *P* < 0.05). Collectively, these data suggest that HSPGs are required for EVT invasion and migration.

### SDC expression in HTR8 EVTs and human placental tissue

**Figure 2:**
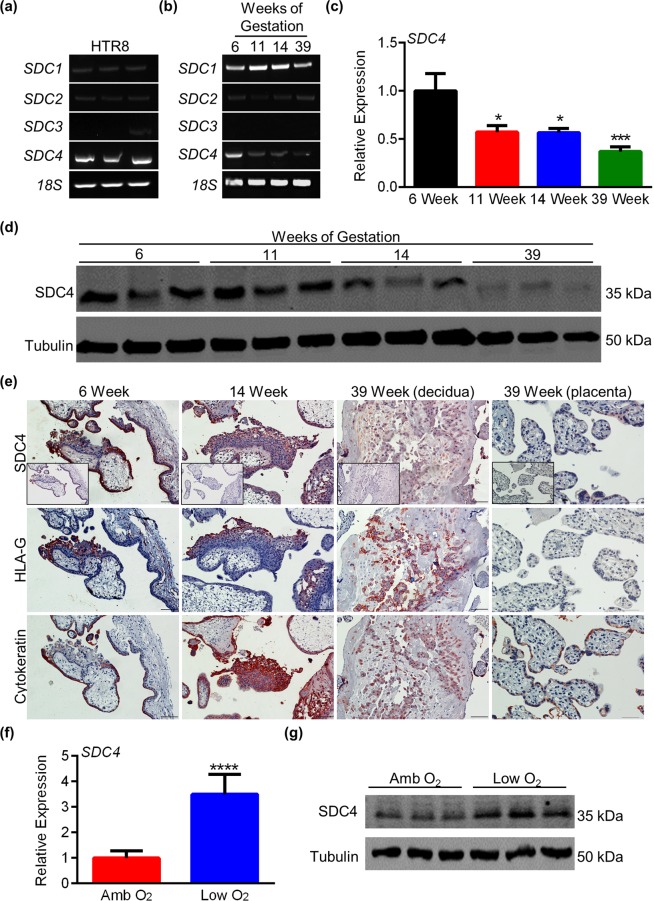
SDC4 expression in HTR8 EVTs and human placental tissue. RT-PCR analysis showing expression of *SDC1*, *SDC2*, *SDC3*, *SDC4*, and *RNA18SN1* (*18S*) in (**a**) HTR8 EVTs and (**b**) human placenta at 6, 11, 14, and 39 weeks of gestation. (**c**) Quantitative RT-PCR analysis of relative *SDC4* expression in lysates of human placental tissue at 6, 11, 14, and 39 weeks of gestation. (**d**) Western blot showing SDC4 protein expression in lysates of human placental tissue at 6, 11, 14, and 39 weeks of gestation. Tubulin was used as a loading control. (**e**) Immunohistochemistry of SDC4 in paraffin-embedded sections of 6, 14, and 39-week human placenta. Human Leukocyte Antigen-G (HLA-G) and cytokeratin staining denotes EVTs and trophoblasts, respectively. Hematoxylin is used to highlight nuclei. Rabbit IgG control images are located in the bottom left corner of the SDC4 panels. (**f**) Quantitative RT-PCR and (**g**) western blot analysis of SDC4 transcript and protein expression in HTR8 EVTs exposed to ambient (Amb) and low O_2_ conditions. Graphs represent means (SEM). Scale bar = 100 μm. Uncropped images of DNA gels and western blots are provided in Figs S4 and S5, respectively. Values significantly different from controls are indicated with an asterisk (*P < 0.05; ***P < 0.001; ****P < 0.0001).

HTR8 EVTs were examined for expression of *SDC1*, *SDC2*, *SDC3*, and *SDC4*. Using RT-PCR, *SDC4* was prominently detected in HTR8 EVTs. *SDC1* was also detected at low levels. *SDC2* and *SDC3* were either expressed at low levels or not detected. Expression of *RNA18SN1* was examined to ensure that an equal amount of cDNA was present in each sample (Fig. 2a, N = 3). In human placenta, *SDC1*, *SDC2*, and *SDC4* were detectable (Fig. 2b). Interestingly, placental expression of *SDC4* varied throughout gestation. Compared to 6-week placenta, there was a significant decrease in *SDC4* expression at 11, 14, and 39 weeks gestation (approximately 43% decrease at both 11 and 14 weeks (*P* < 0.05) and a 63% decrease at 39 weeks of gestation (*P* < 0.001), Fig. 2c, N = 3 placentas measured in duplicate). At the protein level, SDC4 expression was highly expressed during 6, 11, and 14 weeks of pregnancy. Expression was less robust in placental tissue at 39 weeks of gestation when compared to earlier stages of pregnancy (Fig. 2d, confirmation that antibody detects SDC4 is provided in Fig. S1). To determine which cells in human placenta express SDC4, we performed immunohistochemistry using serial sections of 6, 14, and 39-week placenta. SDC4 was strongly detectable in EVTs, as denoted by HLA-G staining, and within syncytiotrophoblast (representing the outer trophoblast layer in chorionic villi, Fig. 2e). SDC4 was also detectable in villous cytotrophoblasts (the inner trophoblast layer in chorionic villi), albeit at less intensity than other trophoblast lineages, and expression was not consistently observed in the villous core (Fig. 2e**)**. Furthermore, consistent with our previous observations, SDC4 expression decreased in placenta (in both syncytiotrophoblast and EVTs) as gestation progressed. To investigate a possible mechanism to explain why SDC4 expression was higher in early gestation trophoblasts than at term, we cultured HTR8 EVTs in a low oxygen atmosphere, which is more representative of the microenvironment in which trophoblasts are exposed during early gestation^26^. Exposure of HTR8 EVTs to low oxygen conditions for 24 h increased both transcript (Fig. 2f, N = 3, P < 0.0001) and protein levels of SDC4 (Fig. 2g).

### SDC4 promotes HTR8 EVT invasion and migration

**Figure 3:**
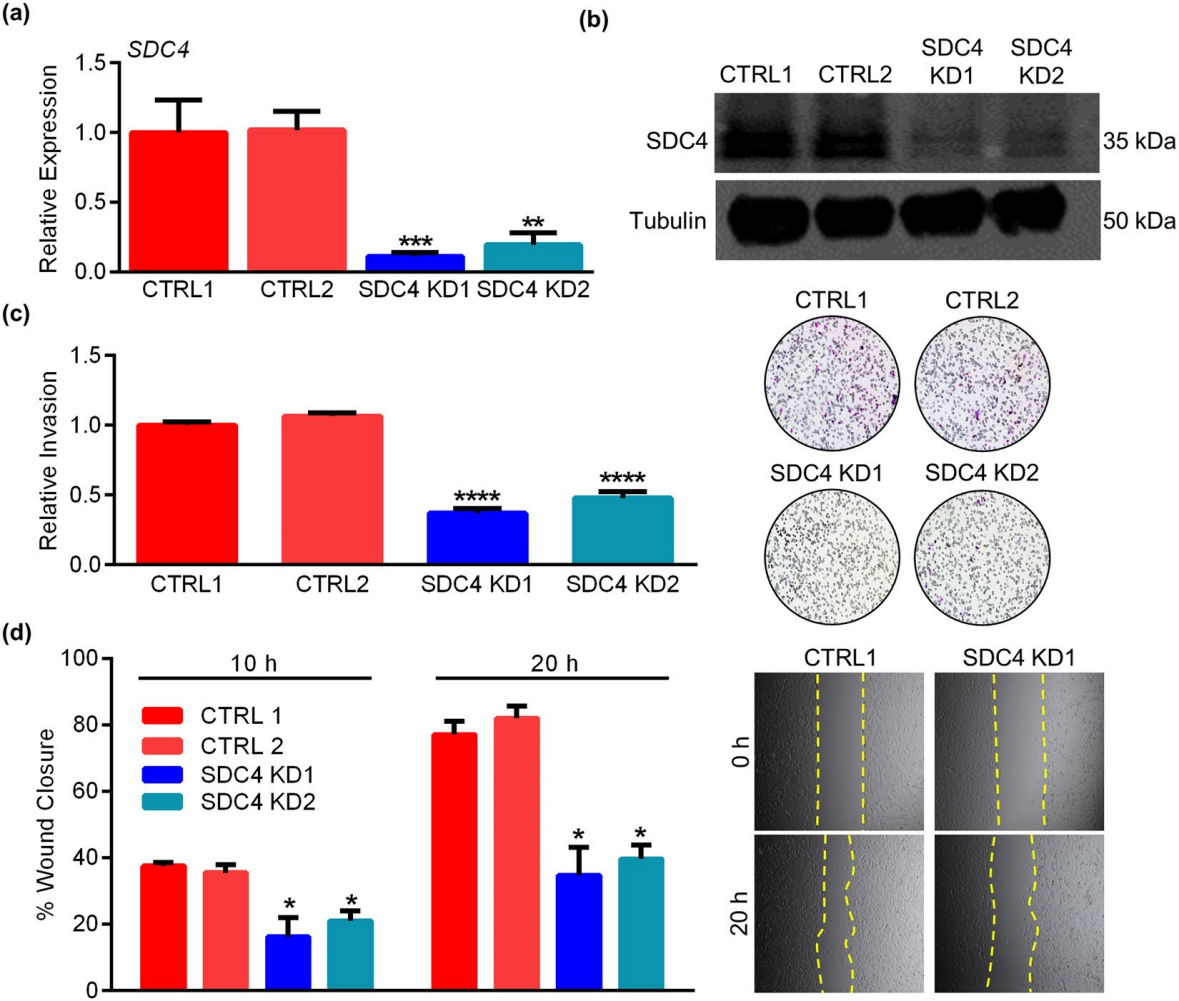
SDC4 knockdown impairs HTR8 EVT invasion and migration. (**a**) Quantitative RT-PCR and (**b**) western blot analysis of SDC4 expression in HTR8 EVTs expressing control shRNAs (CTRL1 and CTRL2) or shRNAs targeting *SDC4* (SDC4 KD1 and SDC4 KD2). Tubulin was used as a loading control for western blots. (**c**) Results from invasion assays showing the relative number of CTRL1, CTRL2, SDC4 KD1, and SDC4 KD2 cells that invaded through Matrigel. Representative images of membranes with invaded cells are shown to the right of the graph. (**d**) Scratch-wound migration assays showing the percent gap closure of CTRL1, CTRL2, SDC4 KD1, and SDC4 KD2 cells, 10 h and 20 h following introduction of a wound. Representative images of the wound at 0 h and 20 h are shown to the right of the graph. The yellow dashed lines denote the wound edge. Graphs represent means (SEM). Uncropped images of western blots are provided in Fig. S5. Data significantly different from controls are indicated with an asterisk (*P < 0.05; **P < 0.01; ***P < 0.001; ****P < 0.0001).

To determine the functional importance of SDC4 in EVTs, we transduced HTR8 EVTs with lentivirus carrying shRNA specific to *SDC4* (SDC4 KD1 and SDC4 KD2). Control cells were transduced with a *scrambled* shRNA (CTRL1), or an shRNA targeted to green fluorescent protein (CTRL2). Using this strategy, we successfully knocked down *SDC4* transcript expression by approximately 90% in SDC4 KD1 (*P* < 0.001) and by 80% in SDC4 KD2 (*P* < 0.01) when compared to CTRL1 (Fig. 3a, N = 10). SDC4 protein expression was also reduced in cells transduced with *SDC4* shRNAs compared to CTRL1 and CTRL2 (Fig. 3b).

To assess whether SDC4 deficiency affected EVT proliferation, control and *SDC4*-deficient cells were cultured for 72 h. Starting at t = 0 h, and every 24 h thereafter, cell numbers were assessed. Compared to CTRL1 and CTRL2, *SDC4-*deficient cells had no change in the rate of cell growth between any of the time-points (Fig. S2a N = 14). To further investigate whether SDC4 deficiency affected EVT proliferation, phospho-histone H3 immunofluorescence was performed, which demarcates cells in the G2/M phase of the cell cycle. There was no difference in the relative percentage of phospho-histone H3 positive cells between control and *SDC4-*deficient cells (Fig. S2b, N = 3), indicating that the rate of cell proliferation was unaffected in EVTs deficient in SDC4.

Since SDC4 is implicated in facilitating cell adhesion to extracellular matrix components, we tested whether *SDC4* deficiency affected the ability of EVTs to adhere to fibronectin or Matrigel. There was no significant difference in cell adhesion to either matrix between control and SDC4-deficient HTR8 EVTs (Fig. S3), suggesting that SDC4 deficiency does not affect EVT adhesion. We next assessed the importance of SDC4 for EVT invasion and migration. Compared to CTRL1 and CTRL2, cells deficient in *SDC4* exhibited a significant decrease in invasion through Matrigel (Fig. 3c, 63% and 52% decrease in SDC4 KD1 and SDC4 KD2 compared to CTRL1, respectively, N = 9, *P* < 0.0001). Likewise, CTRL1 and CTRL2 cells both migrated efficiently following introduction of a wound, closing the initial wound area by 38% and 36% at 10 h, respectively. On the other hand, cells deficient in SDC4 were less efficient in migrating into the wound (16% closed in SDC4 KD1 and 21% closed in SDC4 KD2, Fig. 3d, N = 9, *P* < 0.05). After 20 h, both CTRL1 and CTRL2 cells migrated into approximately 80% of the initial wound area, whereas SDC4 KD1 and SDC4 KD2 managed to close the wound by only 35% and 40%, respectively (Fig. 3d, N = 9, *P* < 0.05). These data indicate that *SDC4* promotes EVT invasion and migration. Since both CTRL1 and CTRL2 behaved similarly in our experiments, we performed all subsequent experiments with only CTRL1.

### SDC4 interacts with PKCα and regulates its phosphorylation

**Figure 4:**
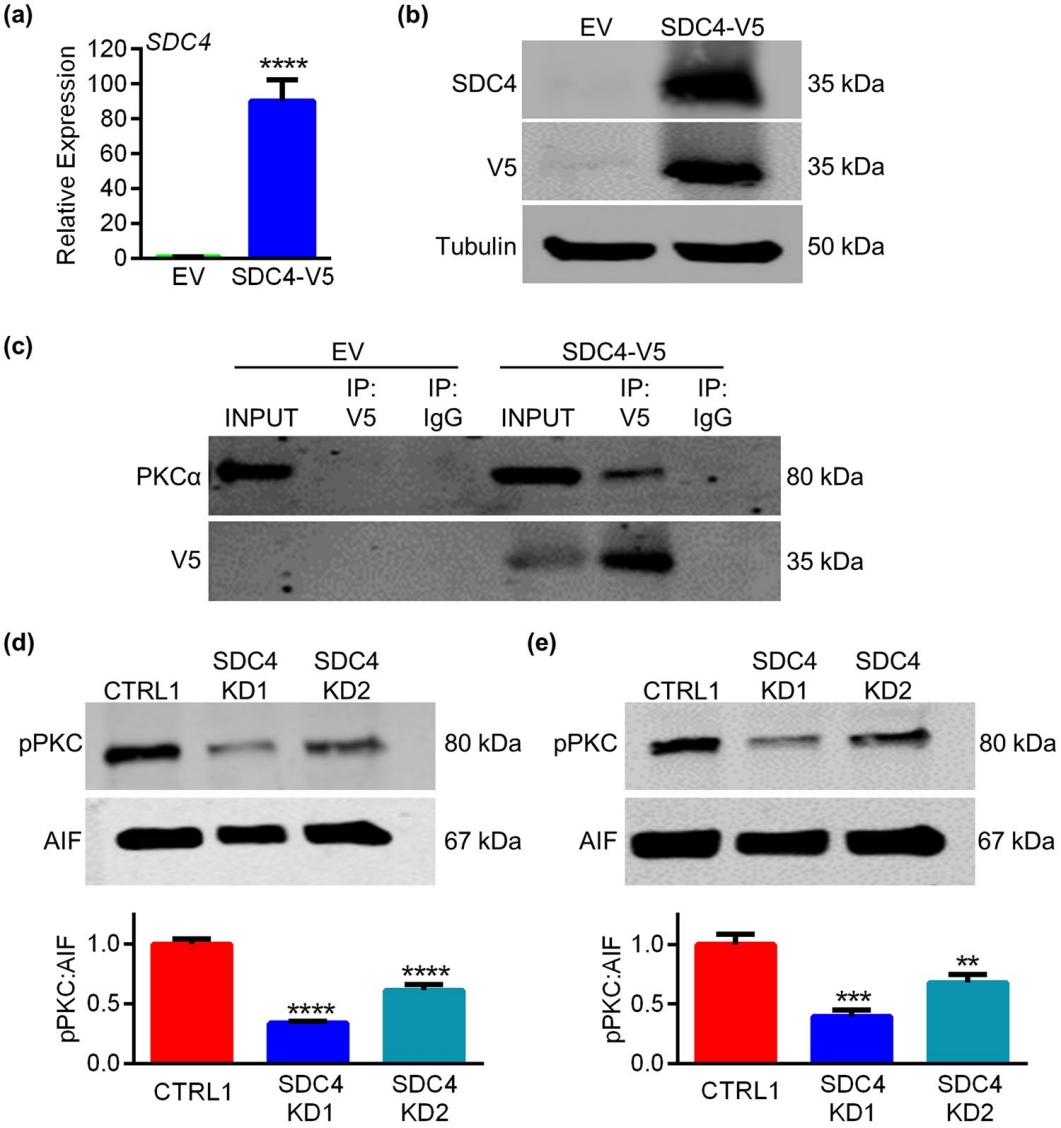
SDC4 coordinates PKC activation. HTR8 EVTs were transduced with an empty vector (EV) or a plasmid encoding V5-tagged SDC4 (SDC4-V5). (**a**) Quantitative RT-PCR analysis showing increased *SDC4* mRNA expression in SDC4-V5 transduced cells compared to cells transduced with EV. (**b**) Western blot showing increased expression of SDC4 and V5 in SDC4-V5 transduced cells compared to cells receiving EV. Tubulin was used as a loading control. (**c**) Co-immunoprecipitation of V5 and PKCα. Input was used as a positive control for western blotting; IgG was used as a negative control for immunoprecipitation. (**d**, **e**) Western blot showing levels of phospho-PKC^Ser660^ (pPKC) in membrane lysates from HTR8 EVTs expressing control shRNA (CTRL1) and cells expressing shRNAs targeting SDC4 (SDC4 KD1 and SDC4 KD2) after plating on Matrigel for 3 h (**d**) or following multiple wounds (**e**). AIF was used as a loading control for membrane lysates. Graphs present results from densitometric analysis comparing pPKC levels relative to AIF of three different western blots. Graphs represent means (SEM). Uncropped images of western blots are provided in Fig. S5. Data significantly different from controls are indicated with an asterisk (**P < 0.01; ***P < 0.001; ****P < 0.0001).

To investigate mechanisms by which SDC4 deficiency impairs motility in HTR8 EVTs, we analyzed activation of PKCα, a putative downstream target of SDC4. To determine whether SDC4 and PKCα interact, we generated HTR8 EVTs either carrying an empty PLX304 vector (EV) or ectopically expressing SDC4 with a C-terminal V5 tag. Cells expressing *SDC4-V5* exhibited a 90-fold increase in *SDC4* expression compared to cells harbouring the EV (Fig. 4a, N = 6, *P* < 0.0001). Ectopic SDC4 expression was confirmed at the protein level and was detected by antibodies for both SDC4 and V5 (Fig. 4b). Immunoprecipitation of V5 from lysates of cells expressing EV and SDC4-V5, revealed that V5 was specifically enriched in the SDC4-V5 cell lysates. Moreover, PKCα was also detected in V5 immunoprecipitated lysates, indicating that SDC4 binds to PKCα in these cells (Fig. 4c). Next, we examined PKC phosphorylation after stimulating migration in CTRL1 and SDC4-deficient HTR8 EVTs. CTRL1 and SDC4-deficient HTR8 EVTs were plated on Matrigel for 3 h (recapitulating the initial stages of a Matrigel-based invasion assay), lysed using subcellular fractionation, and membrane fractions immunoblotted for phosphorylated PKC^Ser660^ (pPKC). Compared to CTRL1, a 67% and 40% decrease in pPKC was observed in SDC4 KD1 and SDC4 KD2 cell lines, respectively (Fig. 4d, N = 3, *P* < 0.0001). To assess the importance of SDC4 for PKC phosphorylation during migration, confluent monolayers of CTRL1, SDC4 KD1, and SDC4 KD2 cells were mechanically disrupted at regular intervals analogous to incorporation of multiple wounds, as previously described^27^. Once again, we observed decreased pPKC levels in membrane fractions of both SDC4-deficient cell lines in comparison to CTRL1 (60% decreased in SDC4 KD1 (*P* < 0.001) and 32% reduction in SDC4 KD2 (*P* < 0.01), Fig. 4e, N = 3). These data suggest that SDC4 interacts with PKCα and facilitates its activation during EVT migration.

### PKC activation promotes EVT motility

**Figure 5:**
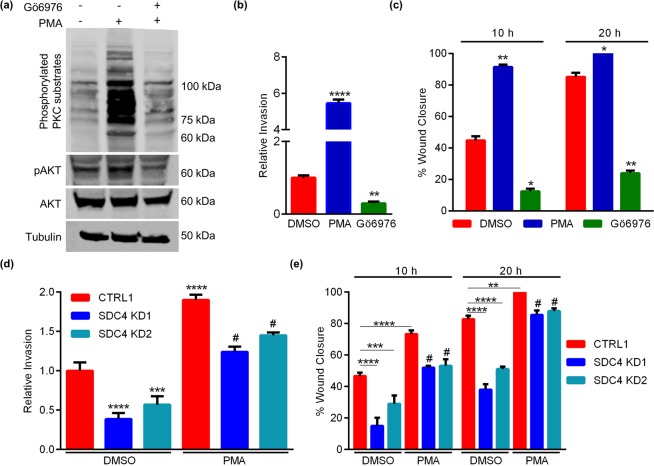
PKC activation promotes EVT motility. HTR8 EVTs were pretreated with DMSO or the PKCα/β inhibitor, Gӧ6976 (10 µM), followed by incubation with the PKC activator, PMA (0.5 µM). (**a**) Western blot analysis of phosphorylated PKC substrates and AKT^Thr308^ (pAKT). Total AKT and tubulin were used as loading controls. (**b**) Number of HTR8 EVTs that invaded through Matrigel following exposure to DMSO, PMA, or Gӧ6976. (**c**) Percent gap closure by DMSO-treated HTR8 EVTs compared to cells exposed to PMA or Gӧ6976, 10 h and 20 h following introduction of a wound. HTR8 EVTs expressing control shRNAs (CTRL1) or shRNAs targeting SDC4 (SDC4 KD1 and SDC4 KD2) were treated with DMSO or PMA, and the number of cells that invaded through Matrigel (**d**) or the percent gap closure 10 h and 20 h following introduction of a wound (**e**) were quantified. Graphs represent means (SEM). Uncropped images of western blots are provided in Fig. S5. Data significantly different from controls are indicated with an asterisk (*P < 0.05; **P < 0.01; ***P < 0.001; ****P < 0.0001). Data significantly different from PMA-treated CTRL1 cells are indicated with a number sign (^#^*P* < 0.05).

To determine the importance of PKC activity for EVT motility, HTR8 EVTs were exposed to the selective PKCα/β inhibitor Gӧ6976 (10 μM). The effectiveness of Gӧ6976 on inhibition of PKC activity was determined by western blotting for phosphorylated PKC substrates using lysates from HTR8 EVTs treated with Phorbol 12-Myristate 13-Acetate (PMA; a potent activator of PKC). Activation of PKC by PMA resulted in strong phosphorylation of PKC substrates. PMA also induced phosphorylation of AKT^Thr308^, a substrate of PKC highly associated with EVT motility^28^. Conversely, pre-treatment of HTR8 EVTs with Gӧ6976 prior to PMA abrogated phosphorylation of PKC substrates and AKT (Fig. 5a). Compared to control cells, cells treated with PMA had increased Matrigel-based invasion capacity (550% increase compared to control cells, *P* < 0.0001), whereas cells treated with Gӧ6976 exhibited significantly reduced invasion (70% decrease compared to control cells, Fig. 5b, N = 6, *P* < 0.01). Likewise, cells treated with PMA had a significant increase in wound closure (10 h following wounding, control cells had closed the initial wound area by 45% whereas cells exposed to PMA had already closed approximately 91% of the gap, Fig. 5c, N = 6, *P* < 0.01). Cells treated with Gӧ6976 had a significantly reduced migratory capacity (33% decrease compared to controls 10 h after wounding (*P* < 0.05), and 61% decrease 20 h after wounding, Fig. 5c, N = 6, *P* < 0.01). These data suggest that PKCα is an important regulator of EVT invasion and migration.

Our next goal was to determine whether PMA-induced activation of the PKC pathway could rescue migration and invasion of SDC4-deficient HTR8 EVTs. After treating CTRL1 and the two SDC4 KD cell lines with PMA, we observed increased invasion of all three lines in comparison to non-treated controls (2-fold increase in invasion in CTRL1 cells exposed to PMA (P < 0.0001), 3-fold increase in invasion in SDC4 KD1, and 2.8-fold increase in invasion in SDC4 KD2, Fig. 5d, N = 3, *P* < 0.05). Similarly, PMA also increased the migration capacity of CTRL1 and SDC4 KD cells. Specifically, a 27% increase was observed in CTRL1 cells (*P* < 0.0001), a 35% increase in SDC4 KD1 cells, and a 23% increase in SDC4 KD2 cells, 10 h post-wounding (Fig. 5e, N = 3, *P* < 0.05). Collectively, these data indicate that activation of the PKC pathway by PMA can partially bypass the requirement for SDC4-mediated PKC phosphorylation, to stimulate EVT motility.

### SDC4 contributes to EVT migration and invasion in response to heparin-binding growth factors

**Figure 6:**
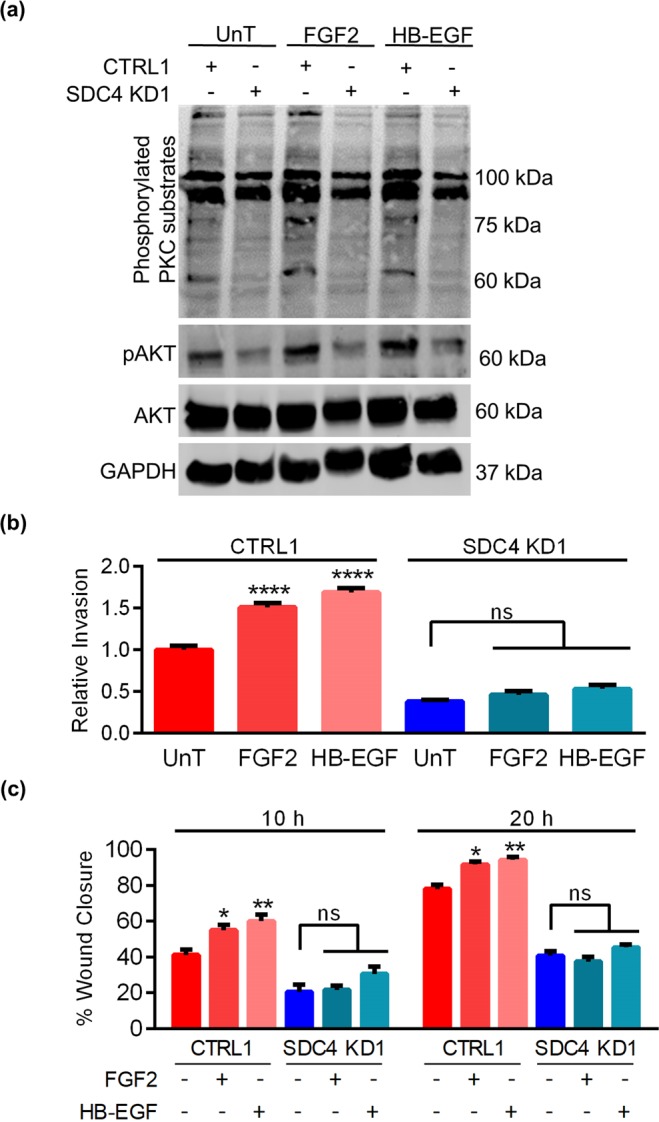
SDC4 promotes EVT motility in response to the heparin-binding growth factors FGF2 and HB-EGF. HTR8 EVTs expressing control shRNAs (CTRL1) or shRNAs targeting *SDC4* (SDC4 KD1) were treated with FGF2 (20 ng/mL) and HB-EGF (20 ng/mL). (**a**) Western blot analysis of phosphorylated PKC substrates and AKT^Thr308^ (pAKT) after exposure to FGF2 and HB-EGF. Total AKT and GAPDH were used as loading controls. (**b**) Number of CTRL1 and SDC4 KD1 cells that invaded through Matrigel following exposure to FGF2 and HB-EGF. (**c**) Percent gap closure by CTRL1 and SDC4 KD1 cells exposed to FGF2 and HB-EGF, 10 h and 20 h following introduction of a wound. Uncropped images of western blots are provided in Fig. S5. Graphs represent means (SEM). Data significantly different from untreated cells are indicated with an asterisk (*P < 0.05; **P < 0.01; ****P < 0.0001).

Our next goal was to assess whether SDC4 contributes to invasion and migration of HTR8 EVTs in the presence of heparin-binding growth factors. The following experiments were completed using CTRL1 and SDC4 KD1 cells. To determine whether specific heparin-binding growth factors activate the PKC pathway by binding to SDC4, we treated cells with either FGF2 or heparin-binding EGF (HB-EGF). FGF2 and HB-EGF increased phosphorylation of PKC substrates and AKT^Thr308^ in CTRL1 cells. However, phosphorylation of PKC substrates and AKT were reduced in SDC4 KD1 cells with and without FGF2 and HB-EGF (Fig. 6a). Invasion of CTRL1 cells was induced by 50% following exposure to FGF2, and increased 70% after treatment with HB-EGF (Fig. 6b, N = 6, *P* < 0.0001). Conversely, FGF2 and HB-EGF had no effect on invasion of SDC4 KD1 cells. Likewise, exposure of CTRL1 cells to FGF2 and HB-EGF significantly increased migration (approximately 14% increase with FGF2 (*P* < 0.05) and 19% increase with HB-EGF (P < 0.01) in wound closure 10 h post-wound), whereas migration of SDC4 KD1 cells was not affected by either growth factor (Fig. 6b and c, N = 6). Our results suggest that SDC4 is required for EVT responses to heparin-binding growth factors.

### Increased SDC4 expression in placentas from preeclampsia

**Figure 7:**
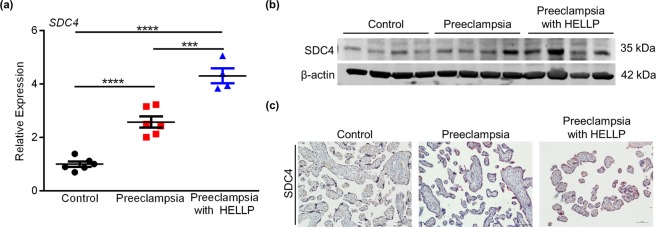
SDC4 expression is increased in placentas from preeclampsia. (**a**) Quantitative RT-PCR analysis showing relative expression of *SDC4* in placentas collected from healthy normotensive pregnancies (control), pregnancies complicated by preeclampsia, and pregnancies from preeclampsia with HELLP. (**b**) Western blot showing expression of SDC4 in lysates of placentas collected from control, preeclampsia, and preeclampsia with HELLP. β-actin was used as a loading control. (**c**) Immunohistochemical analysis of SDC4 in paraffin-embedded sections of placentas collected from control, preeclampsia, and preeclampsia with HELLP. Graphs represent means (SEM). Uncropped images of western blots are provided in Fig. S5. Data significantly different from each other are denoted with an asterisk (***P < 0.001; ****P < 0.0001). Scale bars = 50 μm. *Preeclampsia was clinically diagnosed prior to delivery based on new-onset elevated blood pressure and evidence of proteinuria. HELLP was diagnosed based on clinical measures (e.g. presence of elevated liver enzymes, thrombocytopenia).

SDC4 expression was assessed in placentas obtained from normotensive pregnancies, and placentas from pregnancies complicated by severe preeclampsia with and without the preeclampsia variant HELLP (hemolysis, elevated liver enzymes, low platelets). The gestational age of the pregnancies in which the placentas were collected, as well as parameters used to define preeclampsia, are provided in Table 1. There was a significant increase in *SDC4* expression in placentas collected from early-onset preeclampsia (2.5-fold higher expression compared to controls, Fig. 7a, N = 6, *P* < 0.0001). Even higher expression of *SDC4* was detected in placentas collected from patients with preeclampsia and HELLP syndrome (4.3-fold higher expression than placentas from control pregnancies, Fig. 7a, N = 4, *P* < 0.0001). Both western blot and immunohistochemical analysis showed increased SDC4 in placentas from preeclampsia compared to controls (Fig. 7b,c). Although expression was increased throughout the placenta in preeclampsia with and without HELLP, expression was particularly prominent in syncytiotrophoblast. These data indicate that both SDC4 transcript and protein are elevated in preeclampsia and HELLP.

**Table 1:** Mean (SD) gestational age and blood pressure measurements for patients in which placentas were collected.

Placenta Sample	Mean Gestational Age at Delivery	Number of Samples	Mean Systolic Blood Pressure (SD)	Mean Diastolic Blood Pressure (SD)
Healthy normotensive controls	37.78 (2.87)	6	122.50 (14.18)	72.33 (13.06)
Preeclampsia*	32.04 (2.32)	6	155.33 (14.68)	102.67 (7.71)
Preeclampsia + HELLP*	31.32 (2.32)	4	161.75 (14.06)	102.25 (11.53)

## Discussion

In this study, we found that EVT motility is dependent on the presence of HSPGs. We also discovered that SDC4 is a major HSPG expressed by EVTs. SDC4 is dynamically expressed in human placenta throughout pregnancy and is necessary for mediating interactions with heparin-binding growth factors and facilitating PKCα activation, which in turn promotes EVT motility. SDC4 has previously been shown to have an important role in formation of the labyrinth zone during mouse placentation^29^. To the best of our knowledge, the current study is the first to determine the functional importance of SDC4 in human trophoblast development.

In our initial experiments, we investigated the importance of HSPGs for EVT motility. HSPGs bind extracellular ligands, such as growth factors, chemokines, and matrix molecules, via their heparan sulfate chains. Binding of these ligands to HSPGs modulates cell adhesion, proliferation, migration, invasion, and wound healing in many cell-types, depending on type and concentration of ligand and expression of appropriate HSPGs^30^. Therefore, to determine the importance of HSPGs for EVT motility, binding of heparan sulfate chains to ligands was inhibited by exposing cells to unfractionated heparin and heparinase III. We found that unfractionated heparin and heparinase III reduced HTR8 EVT invasion and migration. Our results are consistent with previous studies investigating the inhibitory effect of unfractionated heparin on hepatocyte growth factor-stimulated invasion of another human EVT line, SGHPL4^31,32^. It should be noted that the effect of fractionated (low molecular weight) heparin on trophoblast motility is more controversial, with some studies reporting that fractionated heparin stimulates EVT migration, and others reporting an inhibitory effect on EVT motility. Unlike unfractionated heparin, fractionated heparin does not displace growth factors captured by HSPGs, or form ternary complexes with growth factors and their receptors^33^. Thus, the biological actions of fractionated heparin on ligand binding to HSPGs differ from unfractionated heparin, which may explain the discrepancies between studies regarding EVT motility following exposure to these heparin species.

SDCs represent the only family of HSPGs that traverse cell membranes, and they are expressed in a cell and tissue-specific context. Previous studies have examined localization of SDC1, SDC2, SDC3, and SDC4 in placental tissues^20,21^. Our data showing that *SDC1*, *SDC2*, and *SDC4* are detectable in placental tissue, and that SDC4 is highly expressed in EVTs, are consistent with these studies. A notable difference in our study was that SDC4 was expressed in syncytiotrophoblast throughout pregnancy rather than only in late pregnancy. Interestingly, we also noted that SDC4 expression was particularly high in placental tissue early in gestation, and that expression of SDC4 decreased as gestation progressed. Although the mechanisms by which placental SDC4 expression changes as pregnancy advances are not known, different oxygen levels at distinct stages of pregnancy may be at least partially responsible. During early gestation before the onset of utero-placental blood flow, the placenta develops in a low oxygen environment ( < 20 mmHg). This low oxygen environment is required for proper placentation and early embryogenesis^34,35^. Once utero-placental circulation is established, the partial pressure of oxygen in placental tissue progressively increases^26^. We found that exposing HTR8 EVTs to low oxygen conditions robustly increased SDC4 expression in these cells. Increased SDC4 expression is observed in other cell types exposed to low oxygen conditions, including colon cancer cell lines^36^ and nucleus pulposus cells^37^, an effect that is dependent on stabilization of hypoxia inducible factor-1. Thus, given our data that SDC4 regulates trophoblast motility and that hypoxic conditions stimulate SDC4 expression, low oxygen-induced SDC4 expression may be important for placental development during early pregnancy.

EVT motility must be carefully orchestrated to ensure proper blood flow to the placenta. Too little invasion may compromise nutrient supply to the baby and can lead to preeclampsia; too much invasion can lead to hemorrhagic complications such as abruption. Consequently, in normal pregnancy, EVTs must integrate cues from the uterine environment, and respond appropriately to ensure that cell invasion proceeds in a sufficient but controlled manner. The serine-glycine motifs in the extracellular domain of SDC4 are decorated with heparan sulfate chains, which facilitate interactions with many extracellular proteins including heparin-binding growth factors. SDC4 also forms ternary complexes consisting of growth factors and their receptors^38,39^. Consequently, SDC4 acts as a central hub that potentiates cellular responses to a variety of extracellular stimuli. Since a major function of SDC4 is to act as a co-receptor for growth factor receptor signaling, we examined the effect of two archetypal heparin-binding growth factors, FGF2 and HB-EGF, on migration of wild-type and SDC4-deficient HTR8 EVTs. SDC4 interacts with both FGF2/FGFR^40^ and HB-EGF/EGFR^41^, and both growth factors are produced by decidual and placental tissues during pregnancy^42^. SDC4 coordinates FGF receptor complex formation by serving as a co-receptor for FGF2 and FGFR, thereby strengthening the intensity and duration of FGF2-mediated signaling events. SDC4 can also function as an independent receptor of FGF2^43^. Similarly, HB-EGF/EGFR can interact directly with amino acids 87–131 in the extracellular domain of SDC4, and disruption in this interaction impairs cell motility in MCF10A breast epithelial cells^41^. In our analysis, we found that FGF2 and HB-EGF increased invasion and migration in wild-type HTR8 EVTs, but not SDC4-deficient EVTs. These results support the paradigm that SDC4 is required for the coordination of EVT motility in response to diverse extracellular ligands, including heparin-binding growth factors.

All SDCs possess a similar structural organization, consisting of an N-terminal ectodomain, transmembrane domain, and C-terminal cytoplasmic domain. The cytoplasmic domains of each SDC possess distinct variable regions that facilitate interactions with a unique assortment of intracellular proteins. The variable region of SDC4 interacts with alpha-actinin, which facilitates linkage with cytoskeletal molecules such as vinculin and zyxin^44^. Moreover, the LGKKPIYKK motif within the variable region of SDC4 interacts with phosphatidylinositol (4,5)‐bisphosphate, and also forms a scaffold for PKCα binding^45^. SDC4-mediated PKCα activation triggers downstream signaling pathways via RhoA and Rac GTPases to facilitate focal adhesion formation and cell migration^46,47^. Thus, SDC4 has been implicated as a key regulator of cell migration in various cell-types^48^. We found that SDC4 directly interacted with PKCα in HTR8 EVTs, and that SDC4-deficient cells had reduced PKC activity and decreased cell motility. Moreover, inhibition of PKCα/β activity abrogated HTR8 EVT invasion and migration, whereas activation of PKC signaling stimulated cell motility. Thus, our data support an essential role of SDC4-PKCα signaling in promoting EVT motility during early placental development.

Defective EVT invasion during the first half of pregnancy is highly associated with early-onset preeclampsia. Reduced EVT invasion compromises blood supply to the placenta, resulting in placental hypoxia, inflammation, and the maternal manifestations of preeclampsia. HELLP is a variant of severe preeclampsia characterized by hepatic dysfunction, hemolysis, and thrombocytopenia^49^. Since our data showed that SDC4 was needed for EVT invasion, we hypothesized that SDC4 expression is decreased in preeclamptic placentas. Interestingly, we observed the opposite effect: placental SDC4 expression was increased in preeclampsia and further increased in placentas of preeclampsia patients with HELLP. It is important to note that all placental tissues collected from preeclampsia and control pregnancies were from third trimester, whereas trophoblast invasion is completed during the first half of pregnancy. Moreover, it was not possible to delineate differences in SDC4 expression specifically in EVTs, since high expression was also evident in syncytiotrophoblast. Thus, it is possible that higher SDC4 expression in preeclamptic placentas may reflect the pathological environment in which the placenta is exposed (e.g. hypoxia), rather than being representative of aberrant SDC4 expression in EVTs during early placental development. This notion is supported by our earlier observation that low oxygen induced SDC4 protein expression. SDC4 is also induced in endothelial cells exposed to inflammation^50^. Inflammation is a prominent feature of preeclampsia and HELLP, so it is possible that the inflammatory environment contributes to higher SDC4 expression in preeclamptic placentas. Interestingly, irregular expression patterns of SDC4 have been described in trophoblastic pathologies characterized by excessive invasiveness, including molar pregnancy and choriocarcinoma^20^. Thus, aberrant SDC4 expression may be an indicator of irregular placentation and could be investigated as a potential biomarker for a variety of pregnancy pathologies.

In conclusion, we found that SDC4 is dynamically expressed in human trophoblast, and coordinates heparin-binding growth factor signaling to activate PKC and promote EVT invasion. Furthermore, SDC4 expression is induced in low oxygen conditions in human EVTs, and is also increased in placentas from preeclampsia, which may be a consequence of the pathological environment in which the placenta is situated. Higher protein expression of other SDCs (notably SDC1) in placental tissue, and lower levels in maternal plasma, have been associated with pregnancies that subsequently develop preeclampsia with or without HELLP^51^. Therefore, in future studies, we will assess SDC4 expression in a larger cohort of placental tissues and in maternal plasma collected from normotensive or preeclamptic pregnancies, to determine whether SDC4 expression can be used to improve detection of preeclampsia prior to when symptoms manifest.

## Materials and Methods

### Tissues, Cells, and Treatments

Paraffin-embedded sections and flash-frozen samples of gestational age 6, 11, 14, and 39-week human placenta, as well as placenta collected from normotensive, preeclampsia, and preeclampsia with HELLP, were obtained from the Research Centre for Women’s and Children’s Health Biobank (RCWIH, Mount Sinai Hospital, Toronto, Canada, http://biobank.lunenfeld.ca). All collections were from caesarean deliveries with appropriate consent, and collections were approved by the Mount Sinai Hospital and University of Western Ontario research ethics boards. The gestational age of the pregnancies in which the placentas were collected, as well as parameters used to define preeclampsia, are provided in Table 1.

HTR8 EVTs, originally derived from immortalization of EVTs derived from explant outgrowths^52^, and commonly used as a model of invasive EVTs, were maintained in RPMI-1640 supplemented with 5% fetal bovine serum (FBS), 100 units/ml penicillin, and 100 µM streptomycin (standard growth media). The cells were kindly provided by Peeyush Lala (University of Western Ontario, London, ON). Human embryonic kidney (HEK)-293T cells were maintained in DMEM supplemented with 10% FBS, 100 units/ml penicillin, and 100 µM streptomycin. Cells were passaged via light trypsinization prior to reaching confluency and were maintained at 37 °C in an atmosphere consisting of 5% CO_2_ for no more than twenty sequential passages. To establish low O_2_ atmospheres (~1% O_2_), a humidified cell culture chamber was placed at 37 °C, and flushed with a gas mixture containing 5% CO_2_/95% N_2_. Levels of O_2_ in the chamber were monitored using a ProOx 110 control device (Biospherix).

The following treatments were applied to cells during experiments: Heparin (10 and 100 µg/ml, H3149, Sigma-Aldrich), Heparinase III (250 and 500 ng/ml, H8891, Sigma-Aldrich), FGF2 (20 ng/ml, 100-18, Peprotech), HB-EGF (20 ng/ml, 100-47, Peprotech), PMA (0.5 µM, 4174, Cell Signaling Technology), and Gӧ6976 (10 µM, 12060, Cell Signaling Technology). When using chemicals reconstituted in dimethyl sulfoxide (DMSO), control cells were treated with the same volume of this agent. All treatments were performed on HTR8 EVTs cultured in standard growth media except for FGF2 and HB-EGF, which were added to cells cultured in serum-free media. Prior to adding FGF2 and HB-EGF, HTR8 EVTs were cultured in serum-free media overnight.

### Matrigel-based invasion assay

Transwells (6.5 mm, 8 µm pore, Greiner BioOne) were coated with growth factor-reduced Matrigel (BD Biosciences, 400 µg/ml diluted in serum free RPMI-1640 medium) for 3 h. Medium was removed prior to plating cells. Approximately 4.0 × 10^4^ HTR8 EVTs were placed on top of the Matrigel, and each chamber was then placed in normal culture medium and incubated for 24 h at 37 °C, 5% CO_2_. After 24 h, excess cells and Matrigel were discarded from the top of the chamber using a cotton swab, and cells that invaded through to the underside of the transwell were fixed in methanol and stained using Diff-Quik (GE Healthcare). Membranes were placed on slides, and invaded cells counted under a microscope.

### Wound assay

Scratch-wound assays were performed to assess cell migration. 2.0 × 10^5^ HTR8 EVTs were plated overnight in 24-well plates under standard growth conditions until a confluent monolayer was formed. Cells were treated with 500 ng/ml mitomycin-C (M4287, Sigma-Aldrich) for 1 h to inhibit cell proliferation. The monolayer was then disrupted by vertically dragging a pipette tip across the cell surface. Images of the wounds were captured at the time of wounding (0 h), and 10 h and 20 h later using a phase-contrast microscope (Leica DFC-295). To ensure images were taken at the same region, a small horizontal wound was introduced and used as a landmark. Between imaging times, cells were incubated at 37 °C, 5% CO_2_. To evaluate the area of wound closure at each time point, images were imported into ImageJ (version 1.5.0), where cell frontiers bordering the wound were traced. The percentage of wound closure was determined using the following equation: [(Ai – At)/Ai] × 100%, where Ai represents the initial area of the wound at 0 h and At represents the area of the wound after incubating for “t” h.

### RT-PCR and Quantitative RT-PCR

RNA was extracted from cells and tissue using Ribozol (Amersham), according to the manufacturer’s instructions, and converted into cDNA via reverse transcription (High Capacity cDNA kit, Life Technologies). cDNA was diluted 1:10, then subjected to conventional PCR or quantitative PCR using primers detailed in Table 2. Conventional PCR was performed using DreamTaq DNA Polymerase (ThermoFisher). Cycling conditions involved an initial holding step (95 °C for 3 minutes), followed by 33 cycles of PCR (95 °C for 30 seconds, 55–63 °C for 30 seconds, and 72 °C for 30 seconds), and a final elongation phase at 72 °C for 12 minutes. Quantitative PCR was performed using a CFX96 Touch (Bio-Rad Laboratories) and SYBR Green PCR Master Mix (PerfeCTa, Quantabio). Cycling conditions involved an initial holding step (95 °C for 10 minutes), followed by 40 cycles of a two-step PCR (95 °C for 15 seconds and 60 °C for 1 minute) and a dissociation phase. Relative mRNA expression was calculated using the ΔΔCt method using both *RNA18SN1* and *YWHAZ* as reference RNAs.

**Table 2:** Forward and reverse primers used for RT-PCR and quantitative RT-PCR (qRT-PCR) amplification.

Gene	Accession No.	Primers (FWD & REV)		Product Size (bp)
*SDC1*	NM_002997.4	FWD	GCTCTGGCTCTGGCTGTG	404
		REV	GTCGTTGAGGCCTGATGAGT	
*SDC2*	NM_002998.3	FWD	AAAACCACAGCAGAGCAAGAAG	476
		REV	AGACGCAGAAGCGTAGTCA	
*SDC3*	NM_014654.3	FWD	CTTCTGCCTCTCCCACTGAC	379
		REV	GCTACCACCTCATTGGCTGT	
*SDC4* (RT-PCR)	NM_002999.3	FWD	GGAGCCCTACCAGACGATGA	470
		REV	AACTCATTGGTGGGGGCTTT	
*SDC4* (qRT-PCR)	NM_002999.3	FWD	ACCAGACGATGAGGATGTAGTG	119
		REV	AAGGGATGGACAACTTCAGGG	
*YWHAZ*	NM_003406.3	FWD	ATGCAACCAACACATCCTATC	178
		REV	GCATTATTAGCGTGCTGTCTT	
*RNA18SN1*	NR_003286.4	FWD	GCAATTATTCCCCATGAACG	123
		REV	GGCCTCACTAAACCATCCAA	

### Western blotting

Cell and tissue lysates were prepared using radioimmunoprecipitation assay (RIPA) lysis buffer (50 mM Tris, 150 mM NaCl, 1% NP40, 0.5% sodium deoxycholate, 0.1% sodium dodecyl sulphate (SDS)) supplemented with protease inhibitor cocktail (Sigma-Aldrich). A bicinchoninic acid assay (Bio-Rad Laboratories) was used according to manufacturer’s instructions to measure protein concentrations. Approximately 50 µg of cell lysate was mixed with 4× reducing loading buffer (0.25 M Tris, 8% SDS, 30% glycerol, 0.02% Bromophenol blue, 0.3 M dithiothreitol), boiled for 5 min, and subjected to SDS-polyacrylamide gel electrophoresis. Proteins were transferred to a polyvinylidene difluoride membrane and probed using antibodies for substrates phosphorylated by PKC (2261, 1:1000, Cell Signaling Technology), phospho-AKT^Thr308^ (13038, 1:1000, Cell Signaling Technology), AKT (4691, 1:1000, Cell Signaling Technology), PKCα (2056, 1:1000, Cell Signaling Technology), SDC4 (HPA005716, 1:250, Sigma-Aldrich), V5 (MA5-15253, 1:1000, Invitrogen), β-actin (47778, 1:1000, Santa Cruz), GAPDH (5174, 1:1000, Cell Signaling Technology), or Tubulin (CP06, 1:5000, EMD Millipore). Membranes were then incubated for 1 h with species-appropriate secondary antibodies, and signals detected using a LI-COR Odyssey imaging system. To assess levels of phosphorylated PKC, membrane fractions were first isolated using a Cell Fractionation Kit, according to the manufacturer’s instructions (9038, Cell Signaling Technology). Western blotting was then carried out as stated above, using a phospho-PKC^Ser660^ antibody (9371, 1:1000, Cell Signaling Technology) and AIF (5318, 1:1000, Cell Signaling Technology). For immunoprecipitated samples, following primary antibody incubation, membranes were immersed with a universal detection reagent (Quick-western kit, LI-COR) in place of secondary antibodies.

### Immunohistochemistry

Serial sections were deparaffinized in Histoclear and rehydrated using increasing dilutions of ethanol washes. Formaldehyde crosslinks were fragmented by placing slides in Reveal Decloaker (Biocare Medical) at 95 °C for 20 minutes. Following rehydration, tissues were treated with 0.3% hydrogen peroxide in methanol to block endogenous peroxidases. Sections were then permeabilized using 0.3% Triton-X and blocked with 10% normal goat serum (Life Technologies). Sections were immersed in primary antibodies specific for SDC4 (36–3100, 1:20, ThermoFisher), HLA-G (21799, 1:100, Santa Cruz Biotechnology), pan-cytokeratin (C11-628608, 1:400, BioLegend), or a rabbit IgG antibody to detect non-specific antibody binding. Subsequently, sections were incubated with species-appropriate biotinylated secondary antibodies, followed by Extravidin peroxidase (Sigma-Aldrich). Placental sections were then treated with an AEC Chromogen Solution (Life Technologies), nuclei counterstained with Hematoxylin, and mounted using Fluoromount-G (Southern Biotech). Sections were imaged using a Nikon DS-Qi2 microscope.

### Transfection, lentivirus production, and transduction

To knockdown SDC4 gene expression, two *SDC4* shRNA constructs encoded in PLKO.1 vectors (shRNA1 – TRCN0000123123 and shRNA2 – TRCN0000299098) were obtained from Sigma-Aldrich. A control PLKO.1 shRNA vector containing an shRNA that does not target any known mammalian transcript, as well as a plasmid that encodes an shRNA targeted against green fluorescent protein, were obtained from Addgene (plasmids 1864 and 12273, respectively). To ectopically express V5-tagged SDC4 in HTR8 EVTs or HEK-293T cells, pLX304 lentiviral constructs containing the complete cDNA sequence of *SDC4* linked to a 3′-*V5* tag were obtained from DNASU (HsCD00439085). Empty pLX304 constructs (EV) were obtained from Addgene (plasmid 25890) and used as a control. Lentiviral plasmids (MD2.G, MDLG/RRE, and RSV-Rev) were used to produce lentivirus, as previously described^53^. Briefly, HEK-293T cells were transfected using Lipofectamine 2000 (Life Technologies) with the EV and SDC4-V5 vectors, or with shRNA-containing vectors and lentiviral plasmids. Lentivirus-containing culture supernatants were collected every 24 h for a total of 48 h. Lentivirus was stored at −80 °C until use. To transduce HTR8 EVTs, cells were exposed to viral particles for 24 h in the presence of 8 μg/ml polybrene. After 48-h infection, transduced cells were selected with either puromycin (3.5 μg/ml; used to select cells transduced with PLKO.1 vectors) or blasticidin (2.0 μg/ml; used to select cells transduced with pLX304 vectors). To avoid any confounding effects of long-term SDC4 deficiency, all experiments involving SDC4 knockdown were completed within one week following incorporation of shRNAs.

### Proliferation assay

Control and *SDC4* knockdown HTR8 EVTs were plated in triplicate wells of a 96-well plate (5 × 10^3^ cells per well) and incubated for 4 h (to enable cells to fully adhere to the wells), 24 h, 48 h, and 72 h. The 4 h timepoint was referred to as t = 0. After each incubation time, cells were washed in PBS, fixed in 4% paraformaldehyde, and stained using crystal violet (0.5% w/v crystal violet, 2% v/v ethanol). After drying, cells were then lysed using 2% SDS. Absorbance was measured at 550 nm.

### Immunofluorescence

Cells were fixed in 4% paraformaldehyde, permeabilized using 0.3% Triton-X and blocked using 10% normal goat serum (Life Technologies). Anti-phospho-histone H3 antibody (1:1600, Cell Signaling) was then added to the wells, followed by goat anti-rabbit Alexa Fluor 488-conjugated secondary antibody. Nuclei were stained using DAPI. Cells were imaged using a Zeiss Axio fluorescence microscope. Quantification of phospho-histone H3 positive nuclei and total nuclei was performed using MATLAB.

### Cell adhesion assay

1% BSA in PBS was placed into 96-well plates for 1 h at 37 °C. Fibronectin (20 ng/ml, Sigma-Aldrich) and Matrigel (5 µg/ml) were then added onto the wells, and incubated for 1 h at 37 °C. Subsequently, the wells were washed, and 1 × 10^5^ HTR8 EVTs were placed onto the wells for 30 minutes or 1 h. Cells were then washed in PBS, fixed using 4% paraformaldehyde, stained using crystal violet, dried, and lysed using 2% SDS. Absorbance was measured at 550 nm.

### Co-Immunoprecipitation

HTR8 EVTs expressing either EV or V5-tagged SDC4 were plated on 100 mm plates and incubated until confluent. Cells were treated with PMA for 20 minutes and lysed on ice using a mild lysis buffer (1% Triton X-100, 150 mM NaCl, 10 mM Tris, 1 mM EDTA, 1 mM EGTA, 0.5% NP-40) supplemented with protease inhibitor cocktail and 100 µM sodium fluoride and sodium orthovanadate. Following sonication, lysates were incubated with 10 µg of mouse V5 (MA5-15253, Invitrogen) antibody or mouse IgG1 (5415, Cell Signaling Technology) as a negative control. Lysates were then incubated with Protein G sepharose beads (P3296, Sigma-Aldrich), and eluted by boiling in 2× reducing buffer. Western blotting was carried out as described above.

### Statistical analysis

Statistical comparisons between two means were tested using Student’s *t* test and statistical comparisons between three or more means were tested using Analysis of Variance, followed by a Tukey’s post-hoc test. Means were considered statistically different if *P* was less than 0.05. GraphPad Prism 6.0 was used for all graphing and statistical analysis. All experiments were repeated at least three independent times.

### Experimental methods guideline statement

All experiments were performed in accordance with relevant guidelines and regulations. All placentas were obtained with informed consent, using protocols approved by the Mount Sinai Hospital research ethics board. The transfer of these tissues for research purposes was governed by a materials transfer agreement and this study was approved by the University of Western Ontario research ethics board.

## Supplementary information

Supplementary Information

## Data Availability

All data generated or analyzed during this study are included in this published article.
