# Supplementary material for: Syndecan-4 regulates extravillous trophoblast migration by coordinating protein kinase C activation
Source: Sci Rep. 2019 Jul 15;9:10175. doi: 10.1038/s41598-019-46599-6 (PMC6629623; doi:10.1038/s41598-019-46599-6)
Supplement: Supplementary file 1 — Supplementary Information [file 41598_2019_46599_MOESM1_ESM.pdf]

Syndecan-4 regulates extravillous trophoblast migration by coordinating protein kinase C activation

Mariyan J. Jeyarajah, Gargi Jaju, Brianna F. Kops, and Stephen J. Renaud

SUPPLEMENTAL INFORMATION

Figure Legends

Figure S1. Verification of SDC4 antibody for use in western blotting. HEK-293T cells were transfected with either an empty vector (EV) control or a plasmid encoding V5-tagged SDC4. Western blot analysis of SDC4 expression is shown. Tubulin was used as loading control. Please note that SDC4 was detected at the expected molecular weight of 35 kDa in lysates of cells transfected with plasmid encoding SDC4.

Figure S2. Effect of SDC4 knockdown on EVT proliferation. (**a**) HTR8 EVTs expressing control shRNAs (CTRL1 and CTRL2) or shRNAs targeting SDC4 (SDC4 KD1 and SDC4 KD2) were plated for 0, 24, 48, and 72 h. Relative cell number was determined by lysing cells after crystal violet staining, then analyzing absorbance spectrophotometrically. Representative images of crystal violet-stained cells 48 h after plating are shown above the graph. (**b**) Percentage of CTRL1, CTRL2, SDC4 KD1 and SDC4 KD2 cells positive for phospho-histone H3 (green). Representative images of cells 48 h after plating are shown above the graph. DAPI (blue) is used to highlight nuclei. Scale bars = 100µm. Graphs represent means (SEM).

Figure S3. Effect of *SDC4* knockdown on EVT cell adhesion. HTR8 EVTs expressing control shRNAs (CTRL1 and CTRL2) or shRNAs targeting SDC4 (SDC4 KD1 and SDC4 KD2) were plated on fibronectin or Matrigel for 30 min (**a**, **b**) or 1 h (**c**, **d**). Cells were stained with crystal violet, lysed, and absorbance measured spectrophotometrically to determine the relative number of cells that adhered to each matrix compared to CTRL1. Graphs represent means (SEM).

Figure S4. Unprocessed DNA gels showing transcript expression of *SDC1*, *SDC2*, *SDC3*, *SDC4*, and *18S*.

Figure S5. Unprocessed western blots.

Figure S1

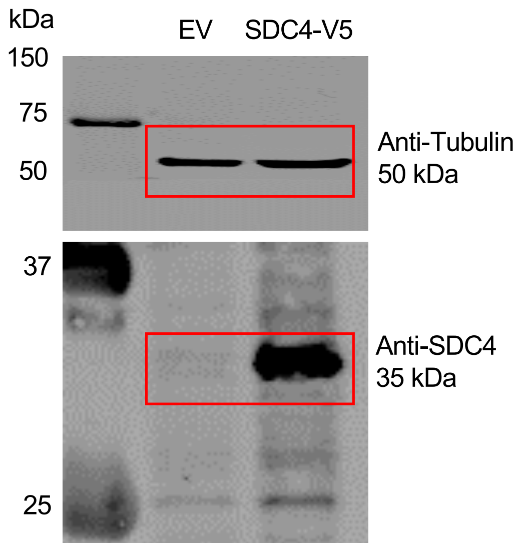
kDa
EV
SDC4-V5
150
75
50
Anti-Tubulin
50 kDa
37
25
Anti-SDC4
35 kDa

Figure S2

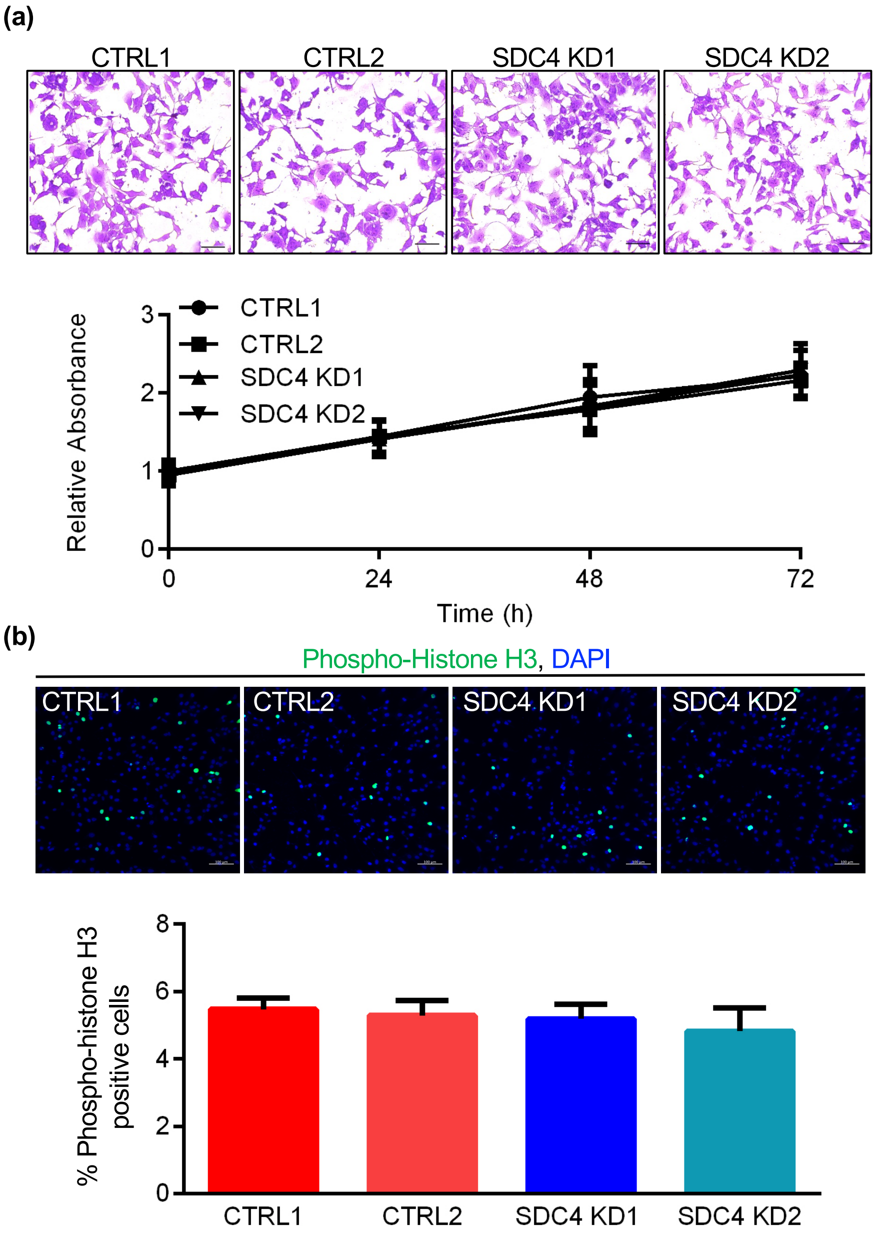

(a)
CTRL1
CTRL2
SDC4 KD1
SDC4 KD2
Relative Absorbance
3
2
1
0
0
24
48
72
Time (h)
(b)
Phospho-Histone H3, DAPI
CTRL1
CTRL2
SDC4 KD1
SDC4 KD2
% Phospho-histone H3 positive cells
8
6
4
2
0
CTRL1
CTRL2
SDC4 KD1
SDC4 KD2

Figure S3

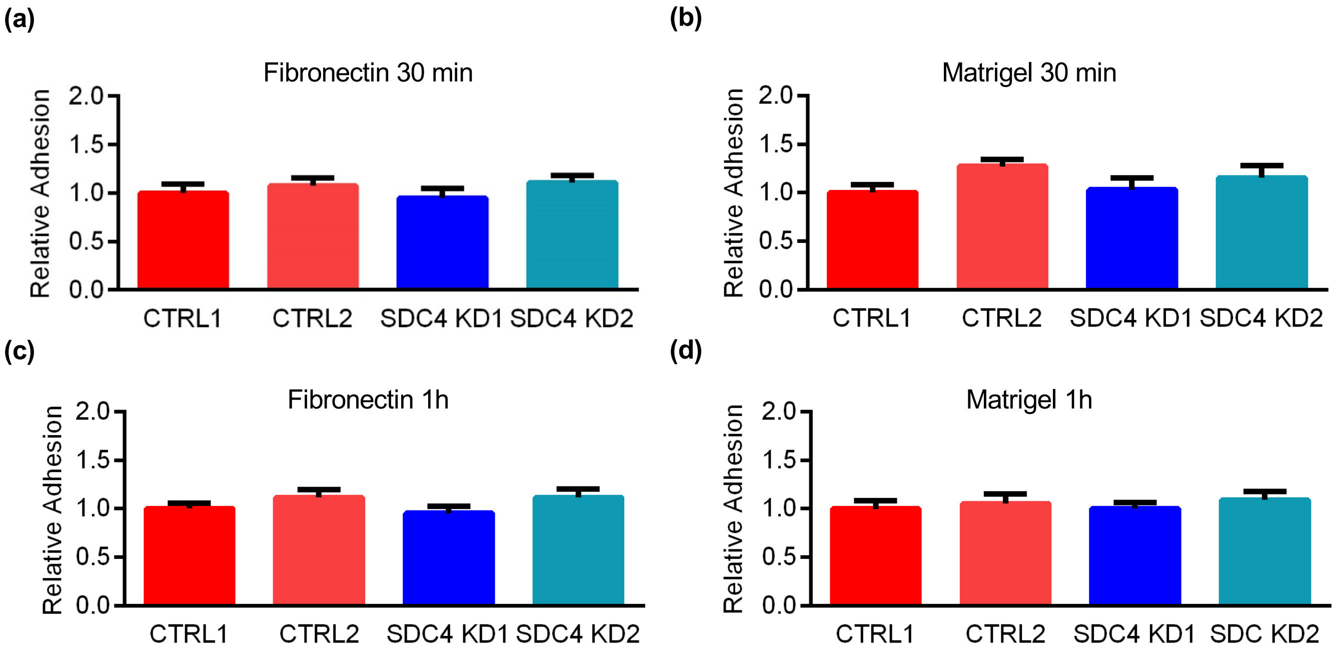
(a)
Fibronectin 30 min
Relative Adhesion
2.0
1.5
1.0
0.5
0.0
CTRL1
CTRL2
SDC4 KD1
SDC4 KD2
(b)
Matrigel 30 min
Relative Adhesion
2.0
1.5
1.0
0.5
0.0
CTRL1
CTRL2
SDC4 KD1
SDC4 KD2
(c)
Fibronectin 1h
Relative Adhesion
2.0
1.5
1.0
0.5
0.0
CTRL1
CTRL2
SDC4 KD1
SDC4 KD2
(d)
Matrigel 1h
Relative Adhesion
2.0
1.5
1.0
0.5
0.0
CTRL1
CTRL2
SDC4 KD1
SDC KD2

Figure S4

Figure 2a

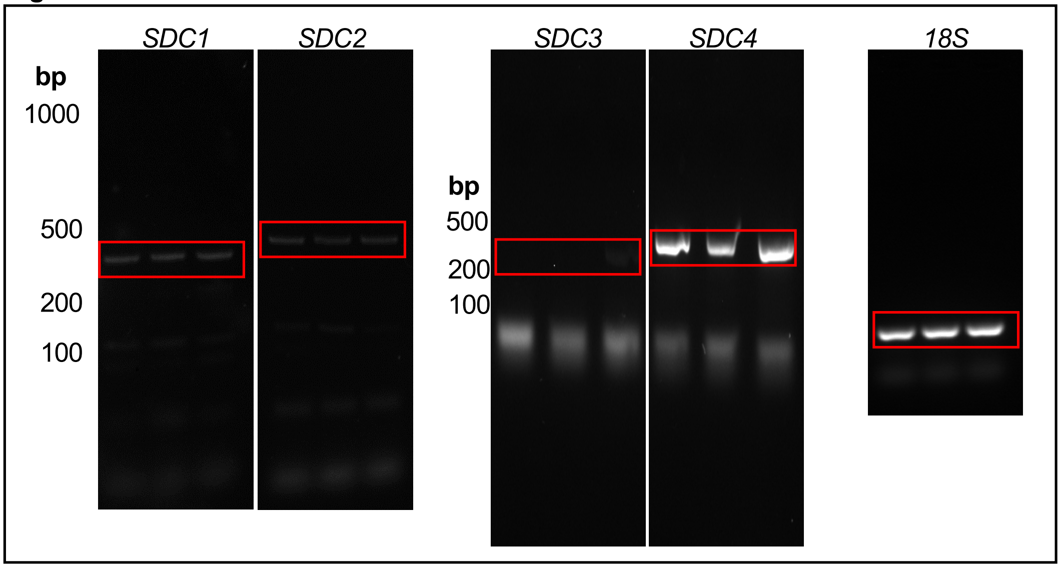

Figure 2b

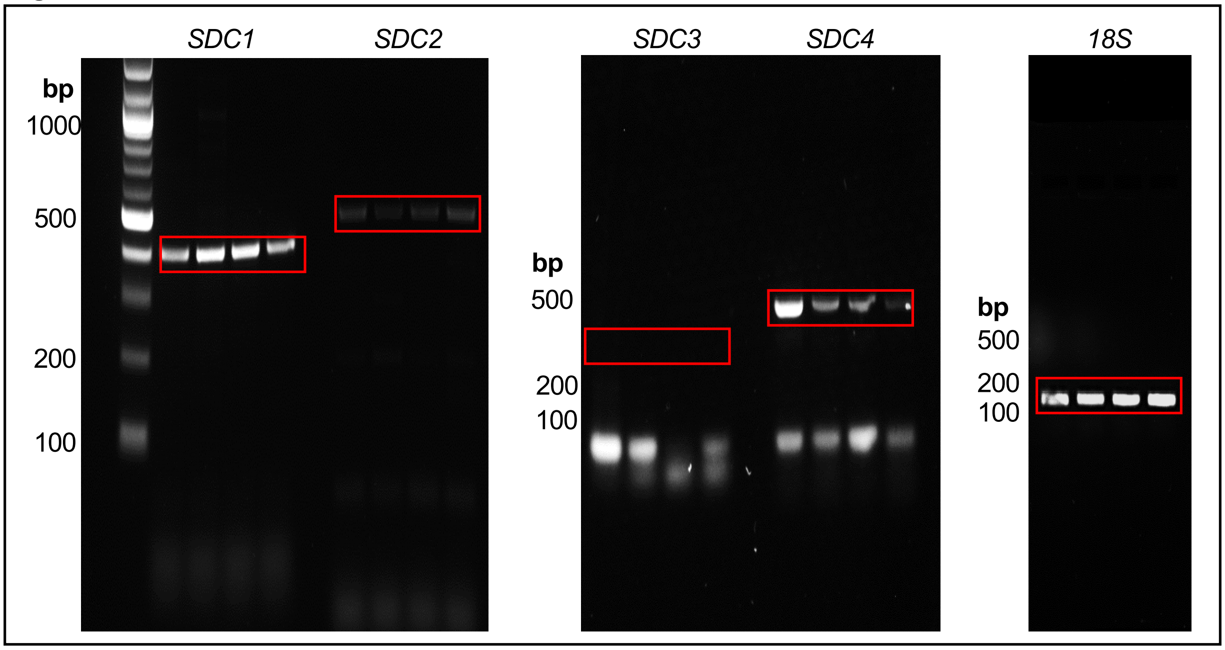

Figure S5

Figure 2d

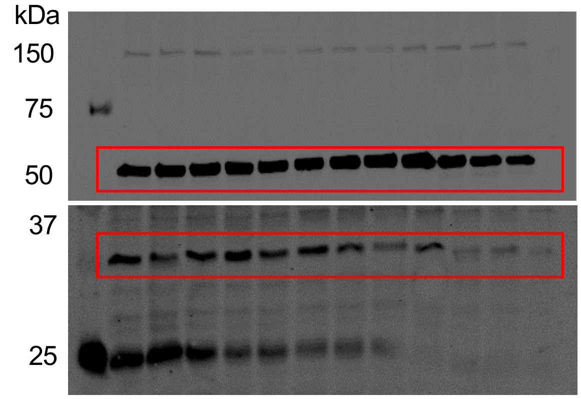

Antibody: Tubulin (Top), SDC4 (Bottom)
SDS-PAGE 10% gel

Figure 2g

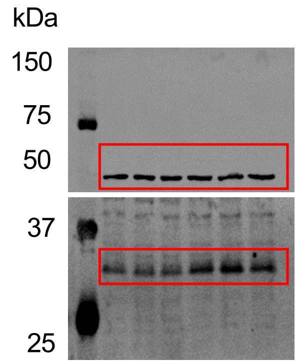

Antibody: Tubulin (Top), SDC4 (Bottom)
SDS-PAGE 10% gel

Figure 3b

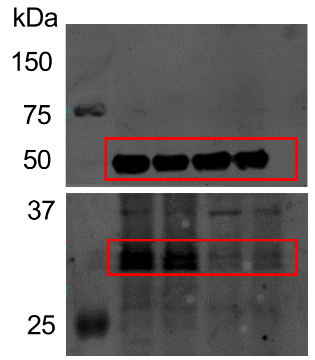

Antibody: Tubulin (Top), SDC4 (Bottom)
SDS-PAGE 10% gel

Figure 4b

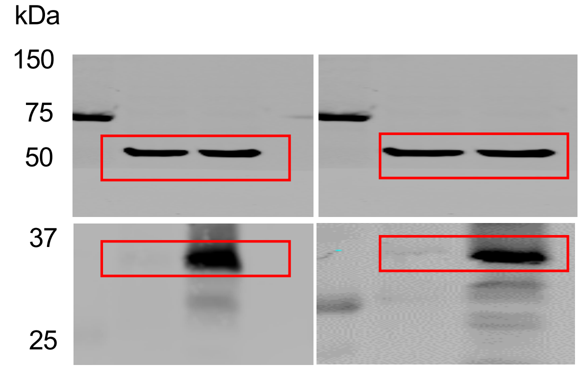

Antibody: Tubulin (Top), SDC4 (Bottom)
SDS-PAGE 10% gel

Antibody: Tubulin (Top), V5 (Bottom)
SDS-PAGE 10% gel

Figure 4c

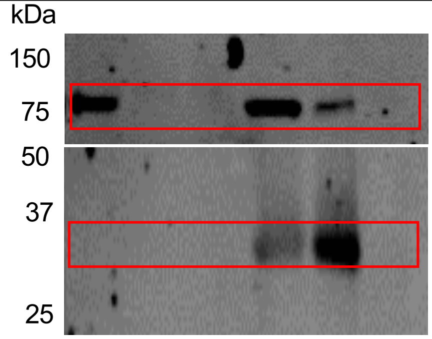

Antibody: PKCα (Top), V5 (Bottom)
SDS-PAGE 10% gel

Figures 4d & e

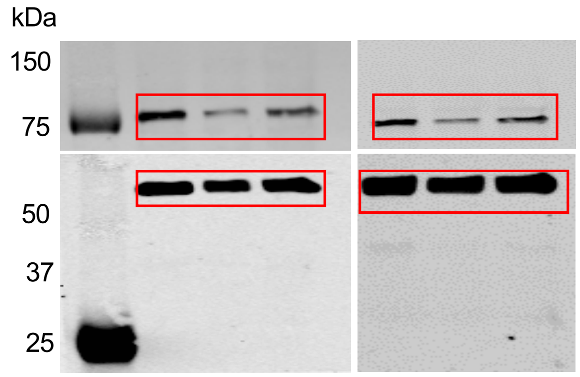

Antibody: pPKC (Top), AIF (Bottom)
SDS-PAGE 10% gel

Figure 5a

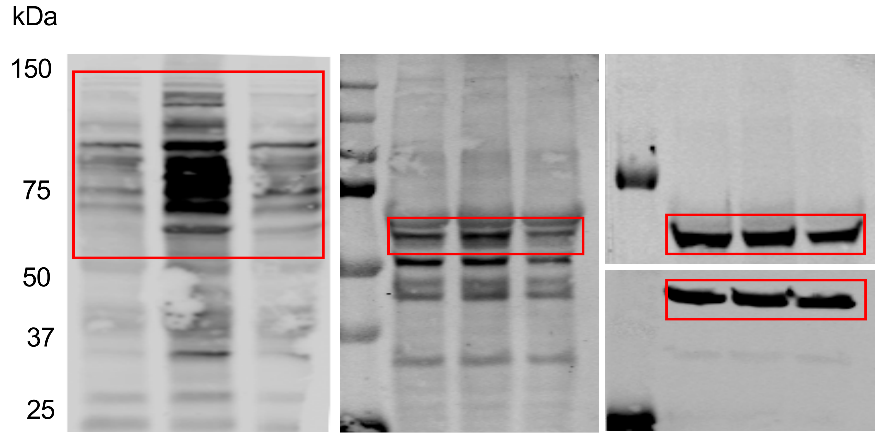

Antibody: Phosphorylated PKC Substrates (Left), pAKT (Middle), tAKT (Top Right), Tubulin (Bottom Right)
SDS-PAGE 10% gel

Figure S5 Cont'd

Figure 6a

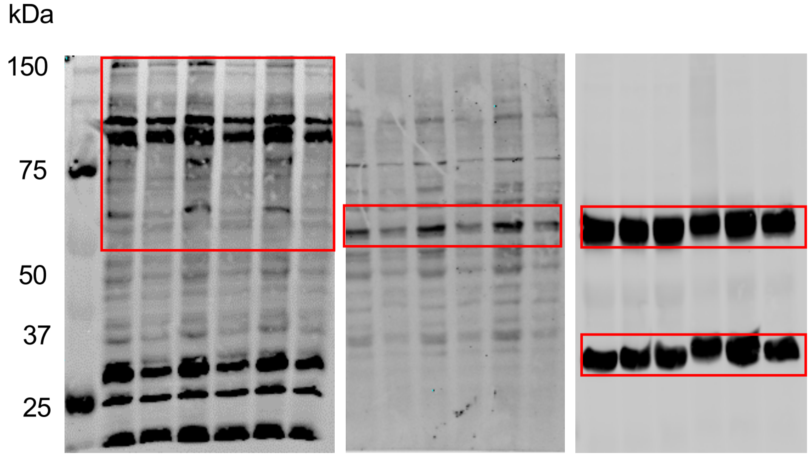

Antibody: Phosphorylated PKC Substrates (Left), pAKT (Middle), tAKT (Top Right), Gapdh (Bottom Right)
SDS-PAGE 10% gel

Figure 7b

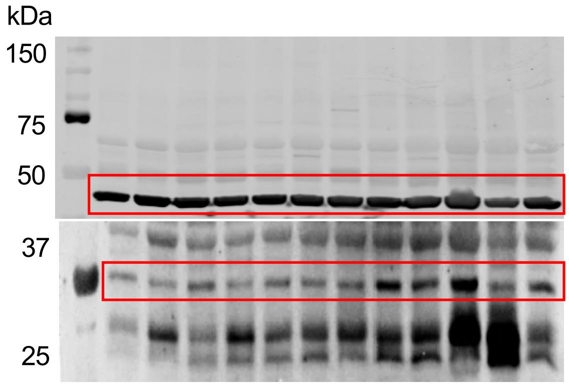

Antibody: β-actin (Top), SDC4 (Bottom)
SDS-PAGE 10% gel
